# The Potential for Information Sharing Between an Electronic Medical Record System and the Moroccan Transplantation and Dialysis Registry: Examining Semantic Interoperability

**DOI:** 10.7759/cureus.57672

**Published:** 2024-04-05

**Authors:** Bassma Bennis, Ghita El Bardai, Basmat Amal Chouhani, Nadia Kabbali, Tarik Sqalli

**Affiliations:** 1 Laboratory of Epidemiology and Research in Health Sciences, Faculty of Medicine, Pharmacy and Dentistry, Sidi Mohamed Ben Abdellah University of Fes, Fes, MAR; 2 Nephrology, Dialysis and Transplantation, Hospital Hassan II, Fes, MAR

**Keywords:** cda, hl7, semantic interoperability, data exchange, moroccan registry for transplation and dialysis (magredial), electronic health record (ehr), electronic medical records (emr)

## Abstract

Background and aim

In 2005, the Moroccan Ministry of Health established Magredial, a registry to track and monitor patients with end-stage renal disease (ESRD), with the aim of improving healthcare outcomes. After achieving initial success, Magredial’s activity decreased, leading to its inactivity by 2015. Currently, efforts are underway to revive Magredial's use. The main goal of this study is to investigate the feasibility of data transfer between the electronic medical records (EMRs) of Hassan II Hospital of Fes, Morocco, and the registry by achieving semantic interoperability between the two systems

Materials and methods

The initial phase of this study involved a detailed review of existing literature, highlighting the importance of registries, especially in nephrology. This part of the study also aims to emphasize the role of semantic interoperability in facilitating the sharing of data between EMRs and registries. Following that, the study's second phase, which centered on the case study, conducted a detailed analysis of the data architectures in both Magredial and the EMR of the nephrology department to pinpoint areas of alignment and discrepancy. This step required cooperative efforts between the nephrology and IT departments of Hassan II Hospital.

Results

Our findings indicate a significant interoperability gap between the two systems, stemming from differences in their data architectures and semantic frameworks. Such discrepancies severely impede the effective exchange of information between the systems. To address this challenge, a comprehensive restructuring of the EMR is proposed. This strategy is designed to align disparate systems and ensure compliance with the interoperability standards the Health Level 7 Clinical Document Architecture (HL7-CDA) set forth. Implementing the proposed medical record approach is complex and time-consuming, necessitating healthcare professional commitment, and adherence to ethical standards for patient consent and data privacy.

Conclusions

Implementing this strategy is expected to facilitate the seamless automation of data transfer between the EMR and Magredial. It introduces a framework that could be a foundational model for establishing a robust interoperability framework within nephrology information systems in line with international standards. Ultimately, this initiative could lead to creating a nephrologist-shared health record across the country, enhancing patient care and data management within the specialty.

## Introduction

In 2005, the Moroccan Ministry of Health established Magredial, a Moroccan registry for transplantation and dialysis. Magredial aims to track the follow-up of patients with end-stage renal disease (ESRD) and monitor their outcomes regarding access to care, survival, treatment modalities for ESRD (dialysis and transplantation), and comorbidities [1]. In 2008, Magredial achieved its highest patient registration rate through the participation of five Moroccan regions. However, by 2011, only the Grand Casablanca region was conducting new registrations, and Magredial has remained inactive since 2015 [2]. Currently, the Ministry of Health is endeavoring to revive Magredial to fulfill the originally established objectives and accomplish the goals of the comprehensive overhaul of the Moroccan healthcare system that began in 2022 [3]. Automating data exchange between electronic medical records (EMRs) and Magredial is considered one of the solutions to register patients on Magredial easily. The Nephrology Department of Hassan II Hospital of Fes, Morocco manages patient data in the hospital information system. The EMR provides practitioners with detailed histories of patients in the department. Its features include monitoring patients' hospital visits, check-ups, and other medical procedures, as well as keeping track of kidney transplant cases performed at Hassan II Hospital. Therefore, investigating the interoperability between the EMR and Magredial holds substantial importance, especially given the extensive adoption of this EMR in Moroccan hospitals and dialysis centers across the Fes-Meknes region. Healthcare interoperability refers to the ability of different systems and software to communicate and exchange data effectively. This valuable connection provides clinicians access to a patient's medical history and current information, ultimately reducing medical errors and improving the quality of care. Moreover, interoperability helps healthcare organizations reduce medical costs by eliminating the need for repetitive tests and leads to better healthcare policy management. The main objective of this article is to explore the possibility of semantic interoperability between the EMR adopted by the Nephrology Department of Hassan II Hospital and the Moroccan chronic kidney disease and transplantation digital registry, "Magredial."

## Materials and methods

Bibliographical study

Medical registries are databases that gather and archive patients' clinical information, along with treatment details and medical outcomes, to conduct clinical research, ensure quality control, and improve the care provided [4,5]. Several studies have highlighted the significance of medical registries in enhancing patient care across various medical specialties [6,7]. The utilization of clinical registries holds great importance in the realm of nephrology. These registries are crucial in improving the quality of care for patients with kidney disease. They offer national epidemiological data for analyzing trends in disease prevalence. Such epidemiological analyses enable anticipation of healthcare infrastructure needs, thereby enhancing the capacity to effectively meet disease management demands [8,9]. Numerous studies underscore the paramount importance of maintaining high-quality data within medical registries. The recorded information must be reliable and accurate [10]. Healthcare professionals must ensure the uniqueness of registered files to mitigate data entry errors and inconsistencies in the collected information [11]. To surmount these challenges, several studies have advocated for implementing standardized protocols for successful data collection [5,10,11]. These guidelines emphasize the importance of collecting data from original sources, such as patient medical records, to maintain information reliability and quality. They also suggest limiting manual data entry and automating the transfer of information from medical records to register to reduce errors. Furthermore, the guidelines highlight the necessity of clearly defining the semantics of data fields to minimize ambiguity during data collection and verifying the uniqueness of files to prevent any duplication. Interoperability enables efficient data transfer between electronic patient records and medical registries. In this context, interoperability refers to the ability to exchange data between two information systems [12]. It operates at both technical and semantic levels. The technical level encompasses the hardware, network protocols, data formats, and communication interfaces that facilitate seamless system sharing. In contrast, semantic interoperability extends beyond mere data transmission and achieves a deep mutual understanding of the shared information. It focuses on aligning the meanings of data and ensuring its interpretation is consistent and accurate. Semantic interoperability has been the focus of numerous scientific studies. For instance, in the United States, Seth Blumenthal at PCPI Chicago discussed in his article [13] the significance of interoperability between patient records and medical registries for enhancing the quality of care. He introduced the "Registries on FHIR" project, aimed at reducing semantic interoperability constraints between these two information systems. Similarly, Nicholas Nicholson and Andrea Perego of the European Commission's Joint Research Centre [14] emphasized the importance of interoperability between medical registries to facilitate the integration of data from diverse sources. Likewise, in their study conducted as part of the Italy-Switzerland INTERREG Dialysis cooperation project, Vito et al. (2016) [15] highlighted the necessity of making the information systems adopted in dialysis centers interoperable. They also presented "La Dialysis MatLib," a dialysis data library capable of converting and harmonizing raw data from different information systems. Besides, Oniel Delva from Florida's Medicare Quality Improvement Organization (FMQAI) [16] underscored the benefits of automating data exchange between various computerized patient records in dialysis centers and CROWNWeb. CROWNWeb is a data collection and verification platform implemented in dialysis centers by the federal Centers for Medicare & Medicaid Services (CMS) in the United States to monitor chronic kidney failure patients. Through interoperability between these systems, admissions and clinical data are updated in CROWNWeb, simplifying data collection and ensuring accuracy.

Case study

This research study aims to investigate the potential for semantic interoperability between Magredial and the hospital information system implemented at the Hassan II Fès University Hospital. This investigation marks the department's first attempt to undertake such research. Achieving semantic interoperability between these platforms will facilitate the automatic feeding of Magredial from the EMR, ensuring a shared understanding of the semantic meaning of exchanged information. The study described in this article was conducted within the Nephrology Department of the Hassan II University Hospital of Fez, through collaboration between the department and the hospital's IT department.

Magredial

The Magredial (Maroc Greffe Dialyse/Morocco Transplant Dialysis) registry is the fruit of Franco-Moroccan cooperation in 2003-2004 [2]. The primary goal of the registry is to provide an accurate depiction of the epidemiological and socio-demographic situation of ESRD in Morocco. It aims to evaluate the current provision of care for the disease and to forecast future national requirements. Magredial is a web platform that enables healthcare professionals to register all patients with ESRD requiring dialysis/transplantation. They can also declare events for hemodialysis patients, such as a change of dialysis center, a change in treatment, or even a declaration of death, and finally register transplant patients and monitor their health status. The registry has been discontinued since 2015. Consequently, it is not possible to monitor or assess the current epidemiological and socio-demographic situation of ESRD in Morocco using data from a reliable medical registry. However, ESRD is a major public health issue in Morocco. The study conducted by Bahadi et al. (2022) [17] estimates the number of patients on dialysis in Morocco at over 30,000, representing an annual cost of over 3.7% of public health expenditure. In February 2023, it was estimated by the Moroccan Society of Nephrology that the number of cases surpassed 37,000. The Moroccan Ministry of Health would like to reintroduce Magredial to the various nephrology care structures to better monitor ESRD's evolution and improve the quality of patient care.

Electronic Medical Record Adopted by the Department

The electronic medical records centralize all computerized patient records in the Nephrology Department of the Hassan II University Hospital in Fez by collecting administrative and clinical patient data. It tracks daily admissions and patients' state of health and coordinates their care with other hospital departments, such as the medical analysis laboratory, radiology, and billing. It is the primary computerized patient record used in the department. The data stored in the system are reliable, accurate, constantly updated, and collected directly from patients.

Methods

To undertake this study, it was imperative to analyze both systems thoroughly. This involved identifying the standards and data models used by each system. Subsequently, it was essential to carry out data mapping to identify similarities and differences between the two systems. Finally, it was necessary to draw up a data conversion plan for the heterogeneous fields in the two systems and to propose a data model that would not only meet the registry's semantic interoperability requirements but also align with international standards of health interoperability such as the Health Level 7 (HL7) Clinical Document Architecture (CDA) standard.

## Results

The EMR data analysis

The patient's administrative and clinical information is recorded in the computerized patient file. Patients are identified by a permanent patient identifier (PPI), which is automatically generated by the EMR when the patient is admitted. Mandatory data for creating the patient record are surname and first name (stored in a single variable), date of birth, gender (female, male, child), national identifier, address, and phone. The healthcare professional can then complete the medical file, indicating the patient's health insurance organization, city, and postal address. Patient follow-up is ensured by the addition of dated free-text reports. The dated discharge diagnosis is documented upon the patient's release.

Magredial data analysis

Patient Identification

Each patient file has an automatically generated Magredial identifier. According to Magredial's detailed functional specifications, the Magredial identifier formula is as follows: region code + province code + patient ID. Magredial verifies the uniqueness of patient files by combining the national identity number (if available), surname, first name, gender, and date of birth. This check is performed automatically when a patient is added. If a similar patient exists, the patient record will be proposed to avoid the creation of duplicates.

Patients' Clinical Data Collection

Subsequently, the patient record is filled with various data, including administrative information, ESRD context, current care structure, initial clinical data and status, comorbidities, and current treatment.

Event/Transplant Reporting and Follow-Up of Transplant Patients

**Figure 1:**
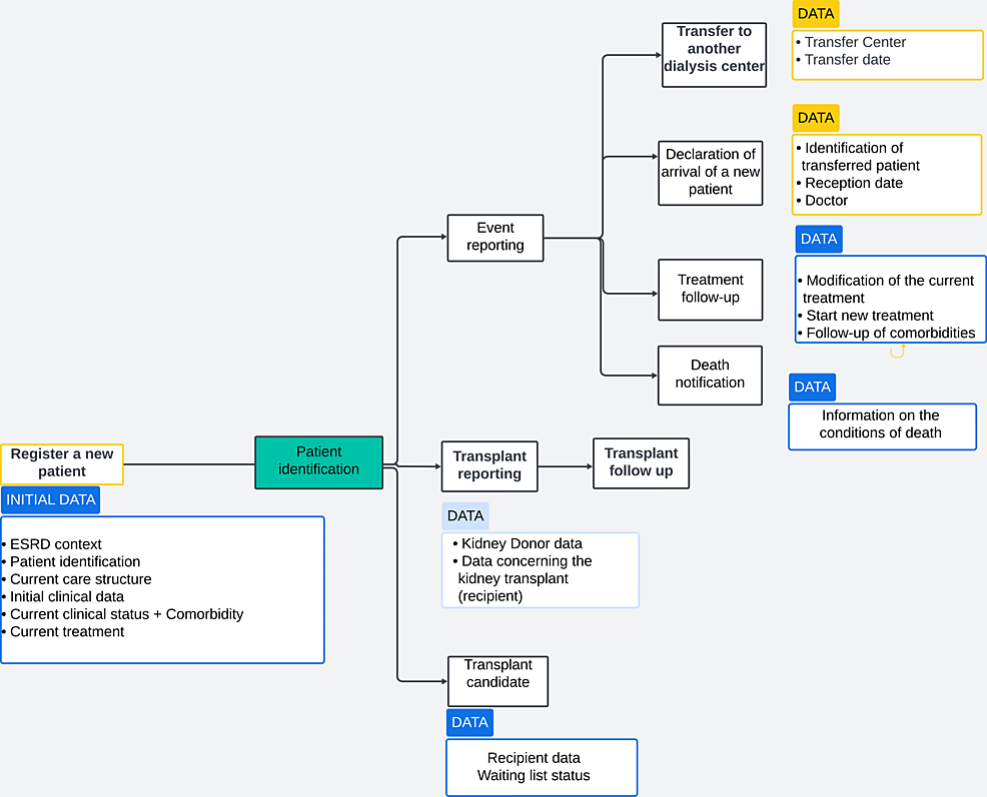
Overview of Magredial register modules and required data inputs ESRD: end-stage renal disease.

From this stage onwards, healthcare professionals can update patient records by registering changes in the treatment modalities, documenting transfers between dialysis centers, and declaring transplant candidates or new transplant patients. Figure 1 below provides an overview of the various modules of the Magredial register as well as the necessary data input for each unit.

Semantic Interoperability Standards

Several global standards have been developed to facilitate semantic interoperability between healthcare systems. Yet, examining the available documentation fails to clarify if the current versions of the platforms adhere to the global standards for semantic interoperability, including HL7 Fast Healthcare Interoperability Resources (FHIR), CDA, Systematized Nomenclature of Medicine - Clinical Terms (SNOMED CT), and Integrating the Healthcare Enterprise (IHE). Furthermore, analysis indicates that the existing data structuring does not align with the structuring advised by these standards.

Data mapping: challenges and difficulties encountered

Data sent from the EMR to Magredial need to be able to enroll new patients and recognize those already registered. Once the patient has been identified, it will be necessary to transfer the information needed to feed and update clinical data, report events (movements between dialysis centers, treatment changes, deaths, etc.), declare and follow up on kidney transplants. Mapping the EMR and Magredial data is crucial for comparing the two datasets and identifying similarities and discrepancies in the information stored in the two systems.

Patient Registration and Identification

The Magredial and the EMR identifiers differ significantly. The medical system is based on an internal personal patient identifier, while Magredial uses the Magredial ID, created from the region code, province code, and an internal patient code. The information required for patient identification on Magredial is either absent or recorded separately from that on the EMR. For example, it is not possible to distinguish between the patient's surname and first name on the EMR, which makes it difficult to determine the corresponding fields on Magredial. Additionally, the hospital system does not provide direct information on the patient's region and province. As a result, Magredial cannot generate the patient identifier adequately from the EMR data.

Collection of Patients' Clinical Data

The hospital system registers most of the information required to feed Magredial. However, the data format and the lack of semantic and syntactic standardization hamper the transfer of information to the registry.

Event/Transplant Reporting and Follow-Up of Transplant Patients

Event reporting requires data to be updated on Magredial. However, nowadays, any change in the patient's situation is made directly on the EMR report. Consequently, it is impossible to identify the correspondence between the EMR and Magredial data.

The comparative analysis of the two systems' data schemas reveals a deficiency in semantic interoperability. The lack of a common language between the two platforms prevented successful data transfer. The absence of EMR data, combined with the lack of variable standardization, is an obstacle to semantic interoperability between the two information systems.

Solutions: patient record template to be implemented on the electronic medical records

It was essential to study the possibility of aligning the department EMR and Magredial. The EMR's flexibility highlighted the possibility of restructuring its patient record to meet Magredial's interoperability requirements. This decision was justified by the feasibility of making this change in-house, unlike Magredial, for which more complex adjustments would be required, involving the Ministry of Health. In particular, this modification involves adding the required data's necessary fields with the same formats and semantics. This restructuring of the patient file will consider all the information currently stored on the digital patient records aiming to preserve the ability to meet the overall service requirements and, specifically, to enhance interoperability with Magredial. Model standardization is imperative for successful data transfer to the registry. Variables integrated into the medical record must have Magredial's data format, authorized values, and validation rules to ensure the consistency, validity, and reliability of exchanges. The model's architectural update aligns with the HL7-CDA standard's guidelines. The choice of this standard is based on its distinctive semantic attributes, widespread adoption within the healthcare IT field, and its proven ability to navigate the complexities inherent in the heterogeneity of diversified information systems.

Patient Registration and Identification

The first step is to complete the administrative information on the EMR and align it with that in Magredial to identify patients correctly. It is important to note that this phase is critical, and any inconsistencies in the data could lead to erroneous search results for patient records in the registry. Consequently, the risk of creating duplicates on Magredial will be significant. The data model that conforms to the CDA standard should include the following information: PPI, gender, last name, first name, date of birth, national identifier, phone number, town of residence, province of residence, region of residence, country of residence, and health insurance company.

Collection of Patient Clinical Data

To feed the registry correctly, it is imperative to standardize the EMR report model and implement semantic and syntactic data management rules. The report will be organized in a manner that comprehensively covers various aspects of patient care and medical history. It will start with the initial data, which includes the patient's medical information upon admission. Following this, the report will centralize the patient's clinical history, detailing dated entries of clinical states to provide a thorough understanding of the patient's current and past medical conditions. The treatment section will trace the patient's history, highlighting past and current interventions. Additionally, the report will include a declaration of transfers or patient reception, which tracks patients' movements within and between dialysis centers. For kidney transplant recipients, specific kidney transplant information will indicate their recipient status and track their post-transplant health. Lastly, if applicable, the report will include a declaration of death to document the end-of-life stage in the patient's medical record.

## Discussion

In 2020, the Kingdom of Morocco initiated several projects as part of the overhaul of the Moroccan healthcare system [3]. Digitizing the healthcare system is one of the four pillars of this overhaul. This project aims to facilitate the collection, processing, and use of healthcare data. Enhancing the semantic data exchange between the department's electronic health record (EHR) and Magredial will facilitate ESRD data collection and meet the overhaul goals. This, in turn, will enable more accurate evaluation and monitoring of the disease's epidemiological situation to enhance patient care. Currently, the absence of reliable data from the Magredial registry complicates the precise determination of ESRD prevalence in Morocco. The literature review conducted in this study fails to yield an accurate and up-to-date national prevalence estimate for ESRD. Furthermore, Asserraji et al. (2015) [18] have also highlighted the shortfall in ESRD data from the national registry. Implementing automatic data transfer will help resolve the obstacles associated with feeding the Magredial, enabling the subsequent establishment of an official Moroccan ESRD register. Furthermore, the data model presented in this study complies with the semantic and syntactic rules specified in the registry's technical specifications. Restructuring patient record data will enable it to comply with Magredial's semantic interoperability requirements. This makes it possible to feed the register automatically via a secure data-sharing interface. The proposed solution for data collection is in line with the recommendations for feeding medical databases derived from the studies conducted by Gopal (2022) [10], Pass (2010) [11], and Williams et al. (2019) [5]. The information collected is reliable and accurate, gathered in real-time from patients. The data transferred are consistent and easily understood by Magredial. Moreover, the uniqueness of Magredial records is ensured by patient identification information, and the risk of error is low, given that manual data entry is limited. Moreover, the suggested framework meets the HL7 standard requirements for patient identification and clinical data standardization. Restructuring the patient record and, in particular, the patient report brings benefits beyond feeding the registry. The data currently recorded in the system require a double analysis effort and are difficult to exploit. Cubelos et al. (2021) [19] corroborate the challenges of extracting non-structured clinical information. According to the study, databases with structured data will empower the data ecosystem and provide better assistance to clinicians and researchers. Also, Ly et al. (2021) [20] support the crucial role of structured records in improving patient history-taking, facilitating communication among healthcare professionals, and enhancing diagnostic accuracy.

Solution's limitations and challenges

Implementing the proposed approach within the existing medical records could prove intricate. This process requires manual restructuring, which is time-consuming [20]. Moreover, the suggested solution demands accurate and comprehensive data entry for the newly created patient records. The proactive involvement and commitment of healthcare professionals are crucial to the success of data entry [21,22]. It will, therefore, be essential to raise their awareness of the importance of exhaustively completing patient records. On the other hand, the possible evolution of the format and value of data on Magredial could hamper semantic interoperability between the two systems. As a result, it will become imperative to monitor Magredial developments from this perspective and to update data on the EMR to bring it into line with new requirements. Beyond the technical aspects, there are significant ethical challenges, notably concerning patient consent for data collection and sharing with Magredial. According to “The White Paper on E-health in Morocco,” respecting confidentiality and security when sharing data is imperative [23]. Data sharing is a significant concern in the Moroccan healthcare sector. Patient data are considered sensitive by the CNDP (Moroccan National Commission for the Protection of Personal Data) [24].

## Conclusions

In the conclusion of our study, the semantic interoperability between the department's EMRs and Magredial systems is crucial for enhancing the ESRD data collection and compilation within the Moroccan context. Our research has illuminated a substantial interoperability gap between these entities, primarily due to discrepancies in patient identification methodologies and significant disparities regarding data formats and structural configurations. The inherent flexibility of the EMR catalyzed the overhaul of the medical record. This redesign preserves the integrity of existing data while concurrently meeting the semantic interoperability criteria of the registry and conforming to the international standards of the HL7 CDA. This framework restructure promises to streamline the data transfer between the two systems and spearhead the development of a comprehensive national ESRD registry. Furthermore, adopting this advanced architectural framework across the spectrum of nephrology information systems is projected to foster an environment of extensive semantic interoperability. This ecosystem will facilitate seamless data interchange between EMRs and Magredial and among diverse nephrology-specific EMRs, laying the groundwork for unified national nephrology-focused EMRs. Such technological advancement is anticipated to substantially enhance the quality of patient care and refine the overarching schema of health data governance.
